# Personality, Anxiety, and Stress in Patients with Small Intestine Bacterial Overgrowth Syndrome. The Polish Preliminary Study

**DOI:** 10.3390/ijerph20010093

**Published:** 2022-12-21

**Authors:** Joanna Kossewska, Karolina Bierlit, Vladimir Trajkovski

**Affiliations:** 1 Institute of Special Education, School Education and Teachers Education, Pedagogical University of Krakow, 30-084 Kraków, Poland; 2 Student Scientific Club of Supporting People with Autism, Pedagogical University of Krakow, 30-084 Kraków, Poland; 3 Macedonian Scientific Society for Autism, Institute of Special Education and Rehabilitation, Faculty of Philosophy, Ss. Cyril & Methodius University in Skopje, 1000 Skopje, North Macedonia

**Keywords:** SIBO, IBS, personality traits, stress, anxiety

## Abstract

Objective: Small intestinal bacterial overgrowth (SIBO) syndrome is associated with depression and anxiety. This study aimed to examine for the first time the correlation between personality traits, situational anxiety, and stress in Polish patients with SIBO. Methodology: This study included 26 patients with SIBO aged 20–35 years and 24 non-SIBO patients aged 20–35 years. The following instruments were used: NEO-FFI Personality Inventory, KPS Sense of Stress Questionnaire, and the anxiety-state subscale from the State-Trait Anxiety Inventory (STAI). Results: Compared to the non-SIBO subgroup, SIBO patients expressed specific patterns of personality traits: higher neuroticism, lower extroversion, and a higher state of anxiety and stress. Unlike the non-SIBO subgroup, stress (total emotional tension, external, and intrapsychic) correlated negatively only with extroversion. Conclusions: Personality is the primary regulator of experience and behavior. The specificity captured in the research is a premise for an in-depth study considering various psychological variables to determine cause-effect relationships.

## 1. Introduction

Increasing evidence of somatic and mental symptoms in the general population, and the strong need for adequate diagnosis and treatment, has drawn attention to the association of gut microbiota with both gastrointestinal and extra-gastrointestinal diseases. Dysbiosis and inflammation of the gut have been linked to several mental health issues, including anxiety and depression, in children [1] and adults [2,3]. A dysbiotic state of the gut microbiota is becoming recognized as an environmental factor that interacts with a host’s metabolism and has a role in pathological conditions, both systemic—obesity, diabetes, and atopy—and gut-related IBS and IBD, although the specific contribution of the gut microbiota to these diseases is unclear [4]. The heterogeneous etiology of metabolic and gastrointestinal diseases has been associated with different microbes, although little information exists about the causal direction of the association [5]. The composition of human microbiota is influenced by host genotype, environment, and diet. Signaling molecules and metabolic products of the microbiota influence several intestinal functions: visceral-sensing, motility, digestion, permeability secretion, energy harvest, mucosal immunity, and barrier effect [6]. One condition could be small intestinal bacterial overgrowth (SIBO) occurring when there is an abnormal increase in the overall bacterial population in the small intestine. However, when diagnosing SIBO, the main criterion is the number of bacteria, not their type. Overgrowth can affect both bacteria that are generally either physiologic or pathogenic. Most often, there is an overgrowth of *Streptococcus, Escherichia coli, Lactobacillus* and *Bacteroides* bacteria [7].

The functioning of the gastrointestinal tract can be impaired by the effect of stress. It is often a trigger for disease, causing relapse or a more severe course of the disease [8], and is strongly related to the (chronic) activation of neurological paths between the hypothalamus, pituitary, and adrenal glands, to produce a cascade of stress hormones and neurotransmitters [9].

Experimental studies indicate that psychological stress can negatively affect the transit time of the small intestine, promote SIBO syndrome, and significantly disrupt the balance of the intestinal barrier [10,11]. Stress, anxiety, and depression can come from many other physical disturbances, such as systemic inflammation or leaky gut and might be related to hormonal changes. The altered microbiome can be one of the most significant contributors to anxiety and/or depression [12].

Studies on stress and anxiety in patients with SIBO are limited and inconclusive. Some studies indicate a strong association with depression, anxiety somatisation, and catastrophising compared to controls with irritable bowel syndrome (IBS) [13,14]. However, others studies show that SIBO patients, compared to IBS subgroups, present lower anxiety, depression, and everyday stress [15].

The chronic activation of the hypothalamic-pituitary-adrenal (HPA) axis may play a crucial role in developing SIBO because the stress response is closely linked to the gut microbiome.

The relation between psychological stress and SIBO based on the neurobiological transfer might be affected by the growth and virulence of bacteria mechanism [16,17,18]. On the other hand, stress might also be the secondary reaction of patients suffering from serious SIBO symptoms. Arguably, a vicious circle of mechanisms is at work here. To predetermine whether there is a primary psychological basis for the occurrence of SIBO, the reference to personality should be made because the Big Five personality model [19] is a strong predictor of illness behavior [20]. The specificity of personality and affective state profile in patients with inflammatory bowel disease [21] was the background to look for specificity in SIBO patients.

Although SIBO patients experience many psychosomatic problems, stress might have also have a significant impact on their mental health.

The present article aimed to describe the peculiar personality pattern and its relationship with stress indicators and the situational state of anxiety in Polish patients with SIBO. The following hypothesis was proposed:

1. Patients with SIBO experience higher levels of neuroticism and lower levels of extroversion than those without SIBO.
2. Patients with SIBO experience a higher situational anxiety-state than those without SIBO.
3. Patients with SIBO experienced higher stress levels than those in the non-SIBO subgroup.
4. The personality and stress correlation in patients with SIBO are significantly different from those in the non-SIBO subgroup.

## 2. Methods

### 2.1. Participants

Fifty people participated in the study, of which 26 were diagnosed with SIBO and 24 without SIBO (non-SIBO control subgroup). 

The SIBO subgroup (13 females, 13 males) aged 21 to 35 years (M = 27.58; SD = 3.56) varied in their education levels (high school: 38.5%, college: 61.5%) as well as place of residence (big city: 76.9%, medium: 11.5% small: 3.8%, rural areas: 7.7%), marital status (married: 50%, unmarried: 50%), and employment status (full-time: 76.9%, student: 19.2%, self-employed: 3.8%).

The non-SIBO subgroup (females: 12, male: 12), aged 21 to 34 years (M = 27.96; SD = 3.81), too, varied in their education levels (high school: 62.5%, college: 33.3%) as well as place of residence (big city: 75%, medium: 12.5% small: 4.2%, rural areas: 4.2%), marital status (married: 50%, unmarried: 41.7%, divorced: 8.3%), and employment status (full-time: 62.5%, student: 25%, self-employed: 12.5%).

### 2.2. Instruments

The following tools were used in the study:

Polish adaptation of NEO-FFI [22], which consists of 60 items, evaluated on a 5-point Likert scale from strongly disagree to strongly agree. Cronbach’s reliability of measurement using the Polish version of NEO-FFI scales ranges from 0.68 to 0.82.

The subscale X-1 of Polish adaptation of STAI [23], The State-Trait Anxiety Inventory [24], to measure anxiety-state. 

The Sense of Stress Questionnaire [25] was used to measure the stress experience’s structure, consisting of 27 statements evaluated on a five-point Likert scale. The questionnaire consists of three subscales: emotional tension, resulting from anxiety and excessive nervousness, with Cronbach’s alpha = 0.708; external stress with Cronbach’s alpha = 0.584; and intrapsychic stress, linked to one’s inability to cope with one’s inner experiences with Cronbach’s alpha = 0.606.

### 2.3. Procedure

This study was conducted in Krakow dietary centres that treat gastrointestinal disorders, including SIBO, within the period since January till March 2022. A hydrogen-methane breath test with lactulose was performed to confirm or exclude the presence of SIBO. Those with a positive breath test result, showing characteristic symptoms of the disorder, such as abdominal pain, bloating, diarrhoea, or constipation, qualified for the SIBO subgroup, and those with a negative breath test result regardless the somatic symptoms were included in the non-SIBO subgroup. Participants did not declare any abdominal surgery, weight loss, nor antibiotic treatment during last two weeks. Participation in this study was voluntary, anonymous, and unpaid.

## 3. Results

Basic descriptive statistics of the studied quantitative variables are presented in Table 1.

Due to the obtained indices of the Shapiro-Wilk test, the values of the skewness of the distribution of the studied variables in the range of +/− 2 (see Table 1), it was assumed that the distribution of the studied variable is not significantly asymmetric with respect to the mean [26]. Such a value was noted for all the studied variables. Therefore, in this chapter, statistical analyses will be performed using parametric tests.

### 3.1. Personality Traits, Situational State of Anxiety and Stress in People with SIBO

Personality characteristics, anxiety, and stress in patients with SIBO is presented in Table 2.

**Figure 1:**
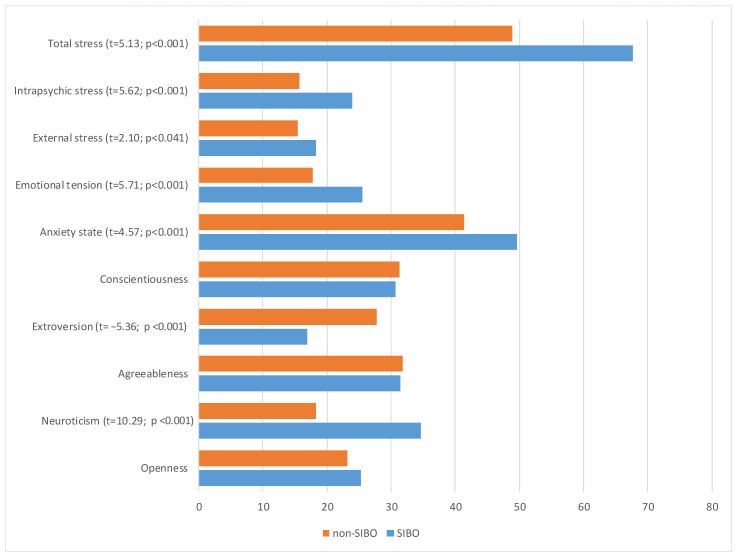
Subgroup differences in personality, anxiety, and stress expressed in means indicators.

In the SIBO subgroup, in comparison to the non-SIBO subgroup, significant differences in personality traits (see Table 2 and Figure 1) were found: higher neuroticism (t = 10.29; *p* < 0.001), and lower extroversion (t = −5.36; *p* < 0.001). The strength of the recorded effects, as measured by Cohen's d coefficient, was very high. The higher anxiety-state was also discovered (t = 4.57; *p* < 0.001) as well as all stress indicators: emotional tension (t = 5.71; *p* < 0.001), external stress (t = 2.10; *p* < 0.050), intrapsychic stress (t = 5.62; *p* < 0.001), and total stress (t = 5.13; *p* < 0.001). The strength of the observed effect for the level of external stress was moderately high, while it was very high for the other three variables.

### 3.2. Personality Stress and Anxiety—State Relationships in SIBO Context

Anxiety state. The relationships between stress and personality traits in the SIBO context are presented in Table 3.

Fisher’s Z tests were performed to verify if the correlations between anxiety state and personality traits met the level of significant differentiation in the subgroups. Pearson’s correlation coefficient was calculated to find the relationship between situational anxiety state and personality traits in the context of SIBO background (Table 3). In non-SIBO subgroup anxiety-state correlated negatively with agreeableness (r = −0.605; *p* < 0.010), extroversion (r = −0.428; *p* < 0.001) and conscientiousness (r = −0.369; *p* < 0.050). This means that increase of situational state anxiety occurred in specific pattern of personality traits (lower agreeableness, extroversion and conscientiousness), however, in SIBO subgroup the increase in situational state anxiety was related only to lower extroversion (r = 0.622; *p* < 0.001).

In the SIBO subgroup, only one personality trait was found to be related to stress. There was a negative correlation between extroversion and total stress (r = −0.405; *p* < 0.050) as well as emotional tension (r = −0.578. *p* < 0.001), and no other relationship between personality and external or intrapsychic stress were found. This means that in SIBO patients, if the level of introversion increases, the total stress, and emotional tension also increases.

In the non-SIBO subgroup, total stress and emotional tension were found to have multidimensional relationship with four personality traits: positively with neuroticism (r = 0.610; *p* < 0.001; r = 0.561; *p* < 0.001) and negatively with agreeableness (r = −0.625; *p* < 0.0001; r = −0.547; *p* < 0.0001), extroversion (r = −0.424; *p* < 0.050; r = −0.568; *p* < 0.001), and conscientiousness (r = −0.425; *p* < 0.05; r = −0.496; *p* < 0.05). External stress positively correlated with neuroticism (r = 0.636; *p* < 0.0001) and negatively correlated with agreeableness (r = −0.640; *p* < 0.0001) and conscientiousness (r = −0.379; *p* < 0.050), but intrapsychic stress was correlated with neuroticism (r = 0.511; *p* < 0.050) and agreeableness (r = −0.565; *p* < 0.01).

The results described above for the SIBO subgroup are interesting; however, only a few subgroup differentiations (SIBO vs. non-SIBO) were statistically significant based on Fisher’s Z test indicators. Significant differences were found in terms of dependency agreeableness and total stress (Z = 2.99; *p* < 0.001), emotional tension (Z = 2.11; *p* < 0.050), and intrapsychic stress (Z = 2.69; *p* < 0.001) as well as in terms of dependency between external stress and neuroticism (Z = −2.74; *p* < 0.001) and agreeableness (Z = 3.23; *p* < 0.0001).

## 4. Discussion

The intestinal microbial ecosystem balance, called eubiosis, is a fundamental concept. The eubiosis/dysbiosis condition of the gut microbiota strongly influences our healthy and disease status [27]. There is microbial imbalance (dysbiosis) in patients with chronic intestinal inflammation and colorectal cancer. A complex interplay between the host, bacteria, and bacterial genes is implicated in the development of these intestinal diseases [28].

Stress has a negative impact on the function of the enteric nervous system, which can consequently lead to the development of dysbiosis, inflammation, or IBS. The gut microbiome is particularly susceptible to stress factors, and the restoration of eubiosis is associated with long term treatment [29]. Gut inflammation is triggered by an imbalanced microbiome, and its effect can be found in various CNS disorders, including, but not limited to, anxiety, depressive disorders, schizophrenia, and autism [30,31]. Because of the excessive translocation of bacteria under stress, it can be inferred that this is one of the factors that may contribute to SIBO. This study aimed to examine the relationships between personality traits, the state of anxiety, and stress in Polish patients with SIBO.

Higher neuroticism was found in the SIBO subgroup, which is related to the overstimulation of the sympathetic nervous system responsible for proper digestion as well as greater vulnerability to stress [32]. Based on the Big Five model, similar conclusions can be drawn because there is a positive correlation between neuroticism and gastrointestinal diseases [33,34,35]. The lower extroversion in the SIBO subgroup may be due to different experiences of positive events, which translates into a different perception of one's dysfunction and experience of somatic complaints. In Polish studies, neuroticism and negative emotionality have been shown to be strong predictors of, among other things, peptic ulcer disease [36], but also other autoimmune diseases such as psoriasis [37].

The correlation between negative emotional states, higher anxiety, and the functioning of the gastrointestinal tract, as well as changes at the level of the gut microbiota in humans and animals, prompted us to consider the occurrence of similar states in patients with SIBO. The hypothesis of a higher state of anxiety and stress in the SIBO subgroup compared to non-SIBO was confirmed. Although the sample size was small, the results were consistent with those of a large group of patients [13]. Recent systematic review studies had shown that medical history and chronic illness are associated with increased anxiety and distress caused by the external situation [38].

Stress defined as a disruption in homeostasis due to external or internal stressors, including environmental or physical and psychological stimuli, may affect the gut microbiota, leading to changes in the host physiology and further increasing disease risk [39]. The stress level in the SIBO subgroup compared with the non-SIBO group was significantly higher, both as total scores and at the level of three indicators: emotional tension, external stress, and intrapsychic stress.

Intestinal complaints caused by the presence of SIBO had a negative impact on the comfort of the subjects' lives; therefore, negative emotional states, tension, or discomfort associated with the symptoms was observed. Other authors also found that stress has an impact on the composition of the microbiota and affects the functioning of the digestive system [40]. They also found a correlation between IBS and increased feelings of stress, anxiety, and the occurrence of sleep problems or worse functioning in daily life, as well as the quality of life [41].

SIBO’s effect of painful sensations, and individual personality characteristics are significant factors that can influence patients’ perception of painful stimuli and their reaction to them [20,42]. Some specific personality traits, such as a negative worldview, might predispose patients to develop psychological comorbidities such as depression, anxiety, and stress. Several studies conducted on adults with gastrointestinal disorders have reported dysfunctional personality characteristics [43,44,45]. Individuals with gastrointestinal disorders exhibit high levels of anxiety and tension, as well as neuroticism, and in stressful situations, they may tend to focus on themselves and their own symptoms, and this promotes increased feelings of negative emotional states [46].

## 5. Conclusions

In conclusion, it can be said that the findings of the study on Polish patients with SIBO is consistent with foreign studies on the correlation of personality traits with gastrointestinal disorders. The present study indicates that patients with bacterial overgrowth in the small intestine showed higher levels of neuroticism and lower levels of extroversion than those without this disorder. The SIBO subgroup had higher situational anxiety and stress, both overall and at the level of the three indicators: emotional tension, external stress, and intrapsychic stress. It was also shown that, in the SIBO subgroup, extroversion was lower than that in the non-SIBO subgroup and correlated with higher stress (particularly emotional tension). Knowledge of the gut-brain axis effects on SIBO pathophysiology and personality can help identify medical and psychological CBT psychotherapy treatments for stress reduction under these conditions [47,48,49].

### 5.1. Limitations of the Study

The study conducted on SIBO patients, implemented on a Polish research sample for the first time, based on purposive sampling, required sampling based on a positive hydrogen test result. Therefore, among the large group reporting to the facility for diagnostic purposes, the subgroup size was significantly limited.

Participants completed psychological questionnaires while waiting for the next hydrogen test result; therefore, they may have experienced situational anxiety in the waiting situation. This procedure may have affected the severity of anxiety, which may have translated into the results of the study.

### 5.2. Future Studies

In future studies, the possibility of interfering factors should be kept at an absolute minimum or subjected to strict control. Such factors as education levels, which may be associated with a higher income and overall socioeconomic status, should be control as a covariate.

Owing to the lack of research on personality traits and the correlation between personality traits and anxiety or stress in patients diagnosed with SIBO, further research in this area is necessary to deepen the topic in question and to draw concrete conclusions. Future studies should explore the activation of the immune response in the mucosa of SIBO-positive IBS patients and healthy controls. There is scope to examine inflammation and immune activation status by determining the imbalance of cytokines and immune cell changes from intestinal biopsies in patients with IBS and SIBO. Therefore, it will be valuable to develop a better understanding of the roles of SIBO and IBS in the pathogenesis of psychological disorders.

## Figures and Tables

**Table 1:** Basic descriptive statistics of the quantitative variables.

	*M*	*Me*	*SD*	*Sk.*	*Kurt.*	*Min.*	*Max.*	*D*	*p*
Openness	24.26	24.00	5.87	0.03	0.06	9.00	37.00	0.99	0.943
Neuroticism	26.76	27.50	9.91	−0.13	−1.20	9.00	42.00	0.95	0.022
Agreeableness	31.58	32.00	5.89	−0.13	−0.16	17.00	44.00	0.99	0.961
Extroversion	22.06	22.50	8.96	0.08	−0.71	6.00	44.00	0.97	0.348
Conscientiousness	31.00	31.00	7.08	−0.31	0.25	11.00	43.00	0.97	0.344
Anxiety state	45.62	46.00	7.56	−0.18	−0.88	30.00	59.00	0.97	0.274
Emotional tension	21.82	23.00	6.15	−0.25	−1.23	11.00	32.00	0.94	0.010
External stress	16.92	17.00	4.88	0.27	−0.29	8.00	29.00	0.98	0.468
Intrapsychic stress	19.90	20.00	6.58	0.27	−0.80	8.00	34.00	0.97	0.154
Total stress	58.64	60.00	15.92	−0.13	−1.03	30.00	85.00	0.96	0.087

Note: *M*, mean; *Me*, median; *SD*, standard deviation; *Sk*, standard deviation—Skewness; *Kurt*—curvature; *Min* and *Max*—the lowest and highest values of the distribution; *D*—the result of the Shapiro-Wilk test; *p*—significance.

**Table 2:** Personality, anxiety, and stress in people with SIBO.

	SIBO Group		Non-SIBO Group				95% CI		
	*M*	*SD*	*M*	*SD*	*t*	*p*	*LL*	*UL*	*d* Cohena
Openness	25.23	6.66	23.21	4.79	1.22	0.227	−1.30	5.35	0.35
Neuroticism	34.58	5.49	18.29	5.70	10.29	<0.001	13.10	19.47	2.91
Agreeableness	31.35	5.10	31.83	6.75	−0.29	0.773	−3.87	2.90	0.08
Extroversion	16.85	7.99	27.71	6.14	−5.36	<0.001	−14.94	−6.78	1.52
Conscientiousness	30.69	5.36	31.33	8.67	−0.31	0.757	−4.81	3.53	0.09
Anxiety state	49.58	6.22	41.33	6.55	4.57	<0.001	4.61	11.87	1.29
Emotional tension	25.54	4.36	17.79	5.23	5.71	<0.001	5.02	10.47	1.62
External stress	18.27	4.50	15.46	4.94	2.10	0.041	0.12	5.50	0.60
Intrapsychic stress	23.85	5.48	15.63	4.80	5.62	<0.001	5.28	11.16	1.59
Total stress	67.65	11.89	48.88	13.98	5.13	<0.001	11.42	26.14	1.45

Note: *M*, mean; *SD*, standard deviation; *t*, results of Student’s *t*-test; *p*, significance; CI [LL, UL], confidence intervals, lower limit and upper limits of the interval; *d* Cohen size effect coefficient.

**Table 3:** Personality traits and stress correlations in two subgroups.

Variables	Anxiety State			Emotional Tension			External Stress			Intrapsychic Stress			Total Stress		
	SIBO (*n* = 26)	Non-SIBO (*n* = 24)	Z	SIBO (*n* = 26)	Non-SIBO (*n* = 24)	Z	SIBO (*n* = 26)	Non-SIBO (*n* = 24)	Z	SIBO (*n* = 26)	Non-SIBO (*n* = 24)	Z	SIBO (*n* = 26)	Non-SIBO (*n* = 24)	Z
Openness	−0.307	−0.088	*Z* = −0.76	−0.113	0.090	*Z* = −0.67	0.309	0.100	*Z* = 0.73	0.083	0.047	*Z* = 0.12	0.114	0.085	*Z* = 0.10
Neuroticism	−0.015	0.285	*Z* = −1.02	0.119	0.561 **	*Z* = −1.71	−0.076	0.636 ***	*Z* = −2.74 **	0.366	0.511 *	*Z* = −0.60	0.183	0.610 **	*Z* = −1.74
Agreeableness	−0.274	−0.605 **	*Z* = 1.39	0.024	−0.547 **	*Z* = 2.11 *	0.212	−0.640 ***	*Z* = 3.23 ***	0.169	−0.565 **	*Z* = 2.69 **	0.167	−0.625 ***	*Z* = 2.99 **
Extroversion	−0.622 ***	−0.428 *	*Z* = −0.90	−0.578 **	−0.476 *	*Z* = −0.47	−0.157	−0.332	*Z* = 0.62	−0.291	−0.374	*Z* = 0.31	−0.405 *	−0.424 *	*Z* = 0.08
Conscientiousness	−0.276	−0.369 *	*Z* = 0.34	0.067	−0.496 *	*Z* = 2.02 *	0.091	−0.379*	*Z* = 1.62	0.064	−0.306	*Z* = 1.26	0.089	−0.425 *	*Z* = 1.80

Note; * *p* < 0.05; ** *p* < 0.001; *** *p* < 0.0001.

## Data Availability

The data presented in this study are available on request from the corresponding author. The data are not publicly available due to ethical reason.
